# COVAX, vaccine donations and the politics of global vaccine inequity

**DOI:** 10.1186/s12992-022-00801-z

**Published:** 2022-03-05

**Authors:** Antoine de Bengy Puyvallée, Katerini Tagmatarchi Storeng

**Affiliations:** 1 Centre for Development & the Environment, University of Oslo, Postboks 1116 Blindern, 0317 Oslo, Norway; 2 London School of Hygiene & Tropical Medicine, London, UK

**Keywords:** Covid-19, Vaccine, Donation, Equity, Foreign policy, Diplomacy, Global health, Public-private partnership

## Abstract

**Background:**

In 2021, donor countries, the pharmaceutical industry, and the COVAX initiative promoted vaccine donation or “dose-sharing” as a main solution to the inequitable global distribution of Covid-19 vaccines. COVAX positioned itself as a global vaccine-sharing hub that promised to share doses “equitably, effectively and transparently,” according to rational criteria overseen by independent scientists. This article provides a critical analysis of the principles and practice of “dose-sharing,” showing how it reveals the politics at play within COVAX.

**Results:**

Donated doses were an important source of COVAX’s vaccine supply in 2021, accounting for 60% of the doses the initiative delivered (543 million out of 910 million). However, donations could not compensate fully for COVAX’s persistent procurement struggles: it delivered less than half of the two billion doses it originally projected for 2021, a fraction of the 9.25 billion doses that were administered globally in 2021. Donor countries and vaccine manufacturers systematically broke COVAX’s principles for maximizing the impact of dose-sharing, delivering doses late, in smaller quantities than promised, and in ad hoc ways that made roll-out in recipient countries difficult. Some donors even earmarked doses for specific recipients, complicating and potentially undermining COVAX’s equitable allocation mechanism.

**Conclusions:**

COVAX’s pivot from global vaccine procurement mechanism to dose-sharing hub can be seen as a “win-win-win” solution for COVAX itself (who could claim success by having access to more doses), for donor countries (who could rebrand themselves as charitable donors rather than “vaccine hoarders”), and for the pharmaceutical industry (maintaining the status quo on intellectual property rights and protecting their commercial interests). Although dose-sharing helped COVAX’s vaccine delivery, its impact was undermined by donors’ and industry’s pursuit of national security, diplomatic and commercial interests, which COVAX largely accommodated. The lack of transparency and accountability mechanisms within COVAX’s overly complex governance structure as a global public-private partnership enabled these practices.

**Supplementary Information:**

The online version contains supplementary material available at 10.1186/s12992-022-00801-z.

*“We don’t want any more promises. We just want the vaccines […] Now is the time for true leadership, not empty promises”* [1].

Dr. Tedros Adhanom Ghebreyesus, WHO Director General, 8 September 2021.

## Background

In September 2021, the CEO of Gavi, which co-leads the global vaccine initiative COVAX, said that “sharing doses with COVAX now represents the single most effective intervention a country can make in terms of halting the circulation of the virus” [2]. His message has been buttressed by dozens of press releases and accompanying social media posts displaying images of smiling donor and recipient delegations alongside branded vaccine shipments and statements from COVAX and donor country leaders mutually praising each other for their shared contributions towards global Covid-19 vaccine equity.

Throughout 2021, COVAX, wealthy governments, and the International Federation of Pharmaceutical Manufacturers and Associations (IFPMA) all promoted vaccine donation as a key solution to what the WHO’s Director General has dubbed a “vaccine apartheid”. COVAX established itself as the go-to global hub for sharing doses “equitably, effectively and transparently,” one that was to be implemented by experts, controlled by independent scientists, guided by ethical principles, and designed to be cost-effective. By capitalizing on a long tradition of framing global health as “evidence-based” and guided by scientific rationality [3, 4], COVAX’s multilateral dose-sharing mechanism was presented as the antithesis of “vaccine nationalism” [5] (that is, unilateral vaccine policies driven by national health security interests) and “vaccine diplomacy” through bilateral donations (deemed to be driven by foreign policy interests).

This article nuances this narrative by analyzing the political interests and diplomatic struggles that have curbed the effectiveness of COVAX’s dose-sharing scheme and limited COVAX’s ability to correct the inequitable global distribution of vaccines: by the end of 2021 only 8.5% of people in low-income countries had received at least one vaccine dose, versus 76–78% in high and upper-middle -income countries [6].

We analyze UNICEF’s publicly available data on global dose donation in 2021 (see methods section below) and show that donating countries and vaccine manufacturers have broken the principles COVAX set for maximizing the impact of vaccine donations. Instead, we argue that stated commitments to vaccine donations and global solidarity have sometimes served as a fig leaf for persistent vaccine nationalism or the pursuit of diplomatic and commercial interests, resulting in donations that came late and in much smaller volumes than promised, in unpredictable shipments, and sometimes even designated for specific recipients, contrary to COVAX’s principles for equitable allocation.

Our findings resonate with the literature showing that global health diplomacy entails potentially contradictory “dual goals” of serving foreign policy interests and strengthening global public health [7–9]. As Feldbaum and Michaud [10] have argued, “countries are increasingly using health initiatives as a means to improve security, project power and influence, improve their international image, or support other traditional foreign policy objectives” (p1). Yet, we seek to move beyond the state-centric bias in the literature on global health security and diplomacy, which leads to insufficient attention to the role of non-state actors and the interplay between national security, diplomatic and commercial interests [11]. As Kickbusch and Holzscheiter [12] insist, “at a minimum, the notion of geopolitics needs to include the power of corporations and private foundations” (p3) to understand future developments in global health security.

From this starting point, we argue that the shortcomings of COVAX and its dose-sharing mechanism cannot simply be attributed to ‘external factors’ (i.e., donor countries’ policies), but also must be understood in relation to the initiative’s own governance structure and internal politics. Although COVAX presents itself as a global coordinating mechanism that goes beyond *national* politics in pursuit of *global* goals, it is in fact a deeply political institution in the sense that it represents certain values, reflects particular ideologies, and preferentially serves some interests over others [4]. As we have argued elsewhere [13], COVAX, and the Access to COVID-19 Tools Accelerator (ACT-A) of which it is part, represent a new, experimental institutional form that brings together established global public-private partnerships (PPPs) into what we have called a “super PPP”. Set up as a weakly institutionalized initiative based on voluntary commitments, COVAX’s overly complex “Russian Matryoshka doll-like governance structure” [13] aimed to cater to widely diverging interests (provide Covid-19 vaccines to rich and poor countries alike) in a context of heightened power asymmetries (an acute global health crisis). However, COVAX is not equipped with the governance mechanisms needed to safeguard the initiative from its partners undermining it, lacks transparency and accountability mechanisms, and, by design, affords too much power to its wealthy government and corporate partners [13, 14]. In the face of these challenges, COVAX has seemingly adopted a pragmatic stance, accommodating powerful partners’ requests and enabling the pursuit of their own self-interests while espousing a rhetoric equity and solidarity – as our analysis of vaccine donation shows. In so doing, COVAX has perpetuated the traditional aid and charity-based model it initially aimed to move away from.

### COVAX's dose-sharing mechanism: an ideal global public health “recipe” for vaccine donation

When COVAX was established in 2020, the COVAX Facility was set up as its procurement arm, managed by Gavi to purchase vaccines on behalf of 177 participating countries through Advanced Purchase Agreements (APA) made with manufacturers while vaccines were still being developed (See Table 1). While so-called “self-financing countries” would pay for their own vaccines, the Facility developed an “innovative financing instrument,” known as the COVAX Advanced Market Commitment (COVAX AMC), to provide access to donor-funded doses to 92 low- and middle-income economies (so called “AMC countries”).

**Table 1:** COVAX glossary [15–18]

**COVAX**: The vaccine pillar of the Access to Covid-19 Tool Accelerator (ACT-A), that aims to accelerate the *development* and *production* of Covid-19 vaccines, and “guarantee fair and equitable access for every country in the world”. Co-led by Gavi, the Vaccine Alliance, the Coalition for Epidemic Preparedness Innovation (CEPI) and the World Health Organization (WHO). **COVAX Facility:** The procurement arm of COVAX that buys vaccines through Advance Purchased Agreements for 177 participating countries, administered by Gavi. **COVAX Advanced Market Commitment (AMC):** The financing instrument that supports 92 low- and middle-income countries (so called ‘AMC countries’) to access donor-funded doses via the COVAX Facility. Has raised over USD 9.7 billion. **Self-financing countries**: Countries that procure vaccine doses through the COVAX Facility, financing the purchase by themselves. **Dose-sharing**: Doses provided to the COVAX Facility to be redistributed to COVAX participants (prioritizing AMC countries). The shared doses should preferably be donated to COVAX, but the initiative may also “consider contributing to the costs of doses at COVAX Facility price” (COVAX principles). **COVAX no fault compensation program:** A liability scheme designed to provide “a lump sum compensation to any individual in the AMC countries who suffers a serious adverse event from any vaccine procured or distributed through COVAX”. **COVAX fair allocation mechanism**: The normative principles that guide the allocation of vaccine doses through COVAX and ensure “fair access and equitable allocation”. **Independent Allocation Validation Group (IAVG):** Composed of 12 independent experts nominated by COVAX co-leaders (Gavi, CEPI, and the WHO). Reviews COVAX allocation proposals and ensures their adherence to the fair allocation mechanism. **Advanced Purchase Agreement:** Public authorities’ contract with a supplier to de-risk a company’s investment by providing upfront payment and guarantee to purchase a good. **Dose-sharing principles:** COVAX developed five principles to maximize the impact of dose-sharing: 1) Doses must be safe and effective; 2) should be donated as soon as possible in 2021; 3) shipped directly from the manufacturer; 4) left unearmarked; and 5) in sufficient and predictable volumes.	

However, the COVAX Facility was not able to procure enough vaccines to reach its objective of vaccinating at least 20% of the population in participating countries. This was in large part because many countries had already secured priority access through their own APAs before COVAX had the financing available to enter into its own deals with manufacturers, or imposed trade restrictions that limited COVAX’s access to vaccine supplies [5, 13]. By the time the first vaccines were approved in late 2020, it was clear that many of these wealthy countries would have access to many more doses than they would need to vaccinate their populations. By December 2020, COVAX had started to work with countries with sufficient vaccine doses and with vaccine manufacturers to share these doses with the COVAX Facility to “complement the early doses procured through the Facility” [15].

COVAX promised that the Facility would ensure that shared doses would be “distributed equitably, effectively and transparently” through the COVAX Allocation Mechanism controlled by independent scientists and guided by normative principles according to a set procedure: The Joint Allocation Taskforce, composed of Gavi and WHO staff, would use an algorithm to develop a “data-driven” proposal for how doses should be allocated (based on population size, operational capacity, and overall vaccination coverage). The proposal would then be reviewed by the Independent Allocation of Vaccine Group (IAVG), a committee of 12 independent experts, which would either approve it or request adjustments needed to ensure that the proposals are “technically informed” (i.e., that they respect the fair allocation principles) [19]. Once approved by the IAVG, the vaccine allocation decision would be signed by the WHO Assistant Director General and the Managing Director of the COVAX Facility.

In addition to equitable vaccine allocation, COVAX’s dose-sharing mechanism promised to be cost-effective by providing: 1) a single contact point for recipient countries; 2) the equipment and on-the-ground assistance necessary for vaccination campaigns; and 3) a liability compensation scheme that would safeguard donors and vaccine manufacturers from legal challenges. COVAX would, moreover, support the world’s poorest countries (AMC-eligible economies) to “optimize readiness for vaccination” and “ensure that ‘no dose sits idle’” [15].

## Results

### Global dose donation to COVAX

Our analysis shows that the practical reality of dose donation has proven to be significantly more complicated than envisioned in technocratic plans. For a start, the very meaning of dose-sharing is contested and in flux.

In a general sense, vaccines can be described as “shared” when these have been procured or purchased by one government but released for distribution in another jurisdiction, without direct payments being involved (though technically, doses shared with COVAX can also be paid for by the initiative) [15]. Throughout 2021, the terms “dose-sharing” and “vaccine donations” were often used interchangeably, though they have distinct ontological meanings [20]. Over time, ‘donation’ gradually supplanted the term ‘sharing’, alongside growing emphasis in COVAX’s communication and in the press on the generosity of the countries providing the doses, with less emphasis on redistributive aspects. The term "donation" has also been used to describe both vaccine doses that have been *pledged* for future delivery and those that have been actually delivered, and a range of scenarios in between [20].^1^

**Fig. 1:**
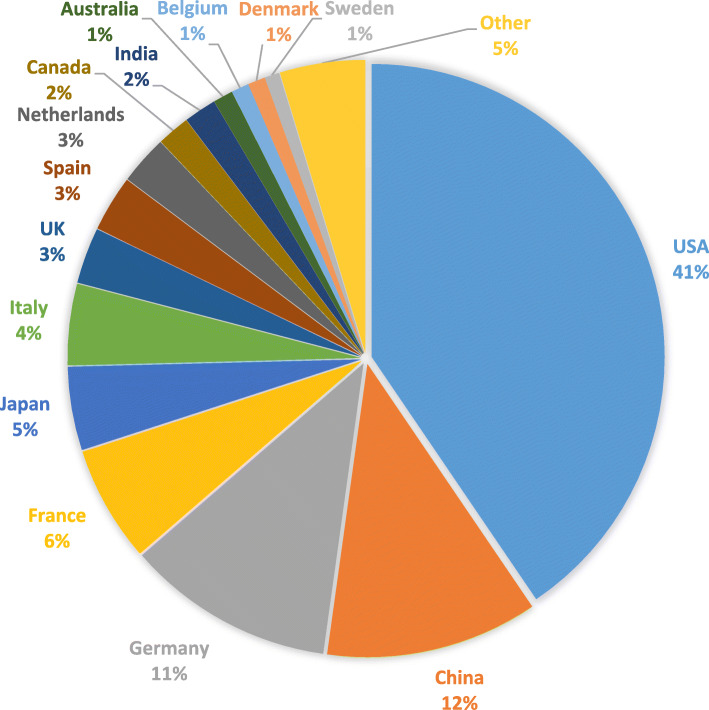
Donated Covid-19 vaccine doses delivered per donor country in 2021 (total = 776 million). Source: UNICEF Covid-19 Market Dashboard [21]. The category “other” includes the 58 donors that delivered less than 5 million Covid-19 vaccine doses for ease of visual representation

If we focus on *actual deliveries*, we see that shared or donated doses were an important source of COVAX’s vaccine supply in 2021, accounting for 60% of the doses the initiative delivered (543 million out of 910 million). However, donation failed to compensate – by far – for the shortcomings of the COVAX Facility’s procurement. As a result, COVAX delivered less than half of the two billion doses it originally aimed to deliver in 2021 (910 million in total, of which 819 million went to AMC countries). This is a fraction of the 9.25 billion doses that were administered globally in 2021 and less than half of the 1.3 billion doses countries pledged to donate by June 2022. COVAX channeled 70% of the 776 million doses donated in 2021. The US was by far the single largest donor of Covid-19 vaccines (41%), followed by China (12%) and Germany (11%). Taken all together, EU countries amounted for a third of the total number of donated vaccine doses delivered in 2021 (Fig. 1).

**Fig. 2:**
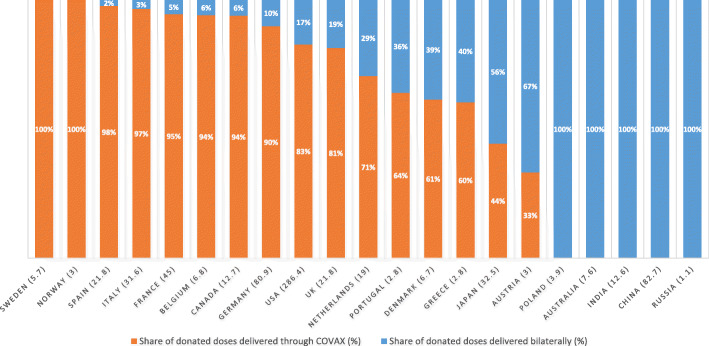
Share of vaccine donations delivered bilaterally vs. through COVAX, by donor country. Source: UNICEF Covid-19 Market Dashboard [21]. Only the 20 largest donors are included in this figure, as well as Russia, which was added due to its vaccine production capacity and much discussed ‘vaccine diplomacy’. In brackets, the total number of doses delivered

Moreover, despite COVAX’s efforts to position itself as a global dose-sharing hub, 30% of donated doses were delivered through bilateral arrangements rather than through COVAX. Countries varied significantly in the extent to which they channeled donations through COVAX in 2021 (Fig. 2). Russia, India, and China did not share any doses with COVAX, donating instead to neighbors or allies. For example, Myanmar and Bangladesh were amongst the top three recipients of donations from both China and India, whereas Russia’s top three recipients were its allied countries Belarus, Syria, and Kyrgyzstan. Similarly, Australia and a handful of EU countries, including Poland, Romania, Hungary, and Latvia also donated exclusively through bilateral agreements rather than through COVAX.

By contrast, Germany, France, Italy, Spain, Belgium, Sweden, Norway, and Canada channeled over 90% of their donated doses to COVAX. The US, the United Kingdom (UK), the Netherlands, Portugal, Denmark, and Japan opted for a middle way, donating to COVAX, but also donating through bilateral arrangements. The Netherlands, for example, started to donate its doses through COVAX only in late November 2021, after it had donated 5.6 million doses bilaterally, with its former colonies Indonesia and Suriname being among the top recipients. Similarly, Portugal channeled 36% of its donations bilaterally to former colonies (Angola, Mozambique, Guinea Bissau, Sao Tome and Principe, and Cabo Verde). Japan stands out as being one of COVAX’s largest financial supporters (with over USD 1bn provided in funding to the COVAX AMC) but donated most of its doses bilaterally to South-East Asian countries. As far as we have been able to ascertain, pharmaceutical companies did not donate any doses to COVAX.

### Dose donation through COVAX – broken promises and violated principles

Our analysis reveals that even for the vaccines that *were* donated to COVAX, donor governments and vaccine manufacturers adhered poorly to the “principles for sharing Covid-19 vaccine doses with COVAX” that COVAX published in December 2020 [15]. To “maximize the impact” of dose-sharing, these principles stipulated that shared doses should be safe and effective, with, at a minimum, WHO prequalification/emergency use listing; be available early in 2021; be of substantive quantity and rapidly deployable, and, crucially, not be earmarked for specific geographies or populations to “facilitate equitable access and in keeping with COVAX’s allocation mechanism” [15].

#### Early delivery of safe and effective vaccines

COVAX’s first principle for shared doses was that they should come early, ideally from the first quarter of 2021. France became the first country to share a shipment of 105,600 doses with COVAX in April 2021, symbolically on ACT-A’s first anniversary. However, although several governments made early pledges to share doses in parallel with their domestic vaccine roll-out, the actual *delivery* of substantial amounts of donated doses to COVAX did not start in earnest until the second half of 2021, when donor countries had largely satisfied domestic vaccine demand for two doses for all adults. Indeed, throughout 2021, COVAX’s leaders grew increasingly frustrated as their call to share doses early were being ignored. In June, Gavi CEO Seth Berkley urged countries “to provide donated doses now and through Q3, while the supply is most limited – and not wait until Q4, when COVAX is forecast to have access to million doses per month from COVAX deals alone” (i.e., from its own procurement) [22] (p7). By September, WHO Director-General Dr. Tedros Adhanom Ghebreyesus (Dr. Tedros) extended a previous moratorium on booster doses until at least the end of the year to enable every country to vaccinate at least 40% of its population [1]. Meanwhile, Seth Berkley reiterated his call to G20 countries to “recommit to COVAX”, stressing that “we need doses, and we need them now.” As he pointed out, only 16.7% of donations pledged to COVAX had been delivered [23].

**Fig. 3:**
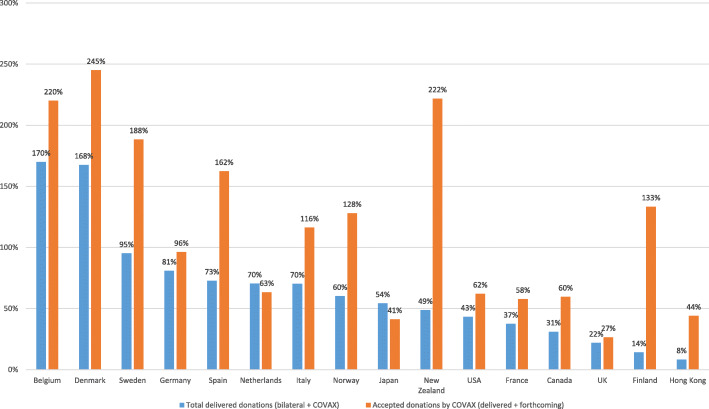
Share of vaccine donation pledges a) delivered (total) and b) accepted by COVAX in 2021, by donor country. Source: Donation pledges retrieved from COVAX [24]; data about delivered doses retrieved from UNICEF Covid-19 Market Dashboard [21]

Countries differed in the speed and extent to which they advanced on the fulfillment of their pledges, with most countries lagging far behind their commitments throughout 2021 (Fig. 3). Many governments claimed to have delivered on their pledges by referring to donations *accepted* by COVAX, even though these might take weeks, and even months to materialize. The gap between ‘accepted donations’ and ‘delivered donations’ (Fig. 3) underscores that most countries’ pledges had not yet been delivered by the end of 2021 and came late. The US, which claimed global leadership in the pandemic response by announcing massive donations, only delivered 43% of its pledge by the end of 2021, though has announced massive deliveries in the first half of 2022. With only 22% of its pledge delivered, the UK stands out as the G7 country that was furthest from delivering on its promises in 2021. Canada (31%) and France (37%) also delivered only a fraction of what they committed to. In fact, only Belgium, Denmark, and Sweden delivered the vaccine doses they committed to donate in 2021. In the end, more than half of vaccine donations to COVAX were delivered during the last 6 weeks of 2021 [21] amidst concerns that the delivery of too many doses at once may overwhelm recipient countries’ “absorptive capacity” and compromise vaccine rollout.

#### Predictable, rapidly deployable, and substantive donations

COVAX’s dose-sharing principles stipulated that donated doses should be of substantive quantity, delivered through predictable shipments, and rapidly deployable. Sharing of doses should be signaled “as early as possible” in the manufacturing process, and dose-sharing countries should facilitate authorizations, “so that doses are shipped directly from manufacturer with universal labelling and packaging, allowing rapid deployment and maximizing shelf-life” [23]. In other words, COVAX asked for larger, predictable volumes to facilitate vaccination campaigns.

In practice, however, according to a COVAX statement from December 2021, “the majority of the donations to date have been ad hoc, provided with little notice and short shelf lives making it extremely challenging for countries to plan vaccination campaigns and increase absorptive capacity” within already stretched health systems [15]. According to WHO’s Dr. Tedros, two thirds of doses donated have had less than 3 months of shelf-life remaining [25]. David Malpass, the President of the World Bank, which manages a financing scheme for COVAX AMC vaccine purchases, has regretted the lack of transparency and predictability of vaccine donations, which has made it difficult to support countries to scale up necessary cold chains or even have the right type of needle available for different vaccine types [26]. Critics have dubbed donations of vaccines with a short shelf life “leftovers” or “vaccine dumping” [27] that is disrespectful of recipient countries and outsources the blame for wasting vaccine doses that would otherwise be destroyed in the donor country.

#### Unearmarked donations

COVAX’s principle of allocating vaccine donations according to pre-defined rational criteria is what sets it most clearly apart from bilateral arrangements, where allocation is assumed to reflect geopolitical factors and national self-interest. An important dose-sharing principle was therefore that doses should *not* be earmarked for specific geographical regions or populations; unearmarked doses would facilitate equitable access, in keeping with COVAX’s fair allocation mechanism [16].

However, some countries appear to have broken this principle. Earmarking of doses is not reported in publicly available databases or in COVAX documents, and COVAX did not reply to any of our requests for data or clarification on this topic (see methods). However, a report in The Lancet [28] states that 75% of the vaccine donations the COVAX Facility received by October 2021 had been earmarked, based on figures obtained from Gavi. Since more than half of COVAX’s supply comes from vaccine donations, if 75% of these doses are earmarked, we can assume that over 42% of COVAX’s total vaccine supply could be earmarked.

In a general sense, earmarking implies that a donor country has indicated the recipient of the donation, thereby directly influencing the allocation of COVAX doses. Yet, earmarking is not a clear-cut concept. According to our interviewees, it can be either *hard* and binding, or *soft* and less committal, and either *specific* (i.e., indicate that doses should or should *not* go to one or more specific country) or *broad* (for example specifying that doses should go to “AMC-eligible countries”). While broad earmarking cannot be ascertained from available data, a large proportion of donated doses going to self-financing (richer) countries may indicate specific earmarking, since poorer AMC countries would likely be favored by COVAX’s fair allocation algorithm. For example, UNICEF’s data showing that between 10 and 23% of Spain, Japan, Canada, and Italy’s donations went to self-financing recipients (against 0 and 3% for other donors) may indicate earmarking for these specific recipients. In the case of Spain, until mid-November 2021, all of its donations through COVAX (8.1 million doses) were delivered to South and Latin-American countries with which it has post-colonial ties. Similarly, until November 2021, all of New Zealand’s donations to COVAX were distributed to six Pacific nations and Indonesia [29].

Although we cannot provide evidence of the extent and nature of earmarking from the available data, it has been of sufficient scale for the IAVG – which oversees COVAX’s fair allocation – to warn in its reports that it “has the effect of complicating allocation decisions, therefore impacting equity and coverage”. The group of independent scientists has recommended that “donor countries to COVAX stop the practice of earmarking doses to selected, pre-specified Participants” [30]. The IAVG “encourages” COVAX to offset the negative effects of earmarking by allocating other, non-earmarked doses to countries left behind, and note that the practice poses “persisting danger to overall equity” [30]. In the absence of data, it is impossible to verify whether this has happened. Nevertheless, because the IAVG approved all the allocations proposed by COVAX, Gavi can conclude in its reports that “the deliberations resulted in consensus on the vaccine allocation decision proposal, with no divergent views recorded”, without even mentioning that earmarking occurs or how it may have been offset [31].

## Discussion

Our analysis shows that while dose-sharing became an important source of vaccine supply for COVAX, it did not compensate for its procurement challenges, compromising COVAX’s ability to reach its original milestones. COVAX was only partially successful in persuading governments with excess vaccine doses to share these with its Facility, with many opting to donate all or some of their surplus vaccines through bilateral arrangements instead of through COVAX. Even for donations to COVAX, donor countries systematically broke COVAX’s dose-sharing principles, failing to deliver on most of their donation pledges within the requested period and then making ad hoc deliveries in ways that have made it exceedingly difficult for recipient countries to roll-out vaccination campaigns. Moreover, some countries’ earmarking of donations has complicated, and possibly compromised, COVAX’s fair allocation mechanism.

Below, we discuss how COVAX partners’ pursuit of national security, diplomatic and commercial interests undermined the effectiveness of COVAX’s dose-sharing mechanism. We then discuss how COVAX’s institutional design and governance largely accommodated these practices as part of a pragmatic attempt to get as many doses as possible within a context of widespread vaccine nationalism globally.

### Unilateralism and national health security interests

Much like wealthy and vaccine-producing countries adopted *unilateral* strategies for vaccine procurement in 2020 to gain prioritized access for their citizens [5], the way they have donated vaccines has also often sought to safeguard national security interests. This finding is in line with two decades of scholarship on global health security showing that the *national* level remains the main referent object of security for Western countries’ engagement with epidemic preparedness and response [32, 33].

While making lofty pledges, donor countries largely held off sharing until doing so would not impact their citizens, with the UK for example, reassuring citizens that “the doses being donated are not needed for the domestic rollout” [34]. Moreover, even if rich countries were to fulfill their donation pledges, they would still be likely to accumulate enormous quantities of surplus vaccines throughout the first half of 2022. Such accumulation in itself constitutes a national preparedness policy that is referred to as “stockpiling,” and occurs at the expense of global vaccination efforts [35].

The US exemplifies how donations can be a way to, at once, demonstrate global solidarity *and* pursue national security interests. In the first half of 2021, the Defense Protection Act was mobilized to oblige American vaccine manufacturers to prioritize deliveries to the US government, resulting in virtually no exports or donations abroad. Even though the US regulatory agency did not approve the AstraZeneca vaccine for domestic use, it was nonetheless stockpiled [36]. Ahead of the G7 meeting in June 2021 – and as the American vaccination campaign plateaued due to vaccine hesitancy – President Joe Biden announced a deal with Pfizer to purchase one billion doses for COVAX “at cost,” including 700 million doses that would count as a “donation”. Unlike other countries, for whom “donation” meant a redistribution of surplus doses, the vast majority of the US “donation” is made up of doses specifically purchased for COVAX, a procurement by proxy of sort. This enabled the US to “donate” without impacting its own vaccine supply, to select the vaccine supplier (Pfizer, a US company) and to maintain strong production capacity for mRNA vaccines (and jobs) at home. In case of crisis or need of extra doses, the Defense Protection Act empowers the US government to redirect the future “donated” Pfizer doses for domestic use. The COVAX donation thus strengthened the US’s preparedness capacity, itself an important national security asset.

### Influence, soft power & “vaccine diplomacy”

That wealthy countries adhered so poorly to COVAX’s dose-sharing principles also reflects that they used their COVAX donations not only to exercise solidarity, but also as a soft power tool to achieve diplomatic recognition and influence [8, 10], so-called “vaccine diplomacy”, and domestic political gain.

During the Covid-19 pandemic, vaccine diplomacy has most commonly been associated with China [37], where it has been seen as “a natural extension of Chinese soft power” [38] that has been used strategically for nation branding, building China’s reputation as a benevolent state and repairing its image following criticisms for its lack of transparency in the early days of the pandemic. Our finding that China donated all its doses bilaterally aligns with previous descriptions of China’s global health diplomacy as being “fundamentally state-centric” [39]. It is also consistent with China’s previous uses of medical diplomacy for hard (material benefits) and soft (image-building) power objectives, for example through sending Chinese medical teams to African countries [40]. In some cases, its bilateral vaccine donations have allegedly come with specific conditions attached. For example, Paraguay’s foreign ministry claims that China offered vaccine doses in exchange for the country breaking its diplomatic ties with Taiwan [41]. However, the scale of Chinese donations (82.7 million doses) is modest compared with the estimated billion doses the country has exported, a third of the doses China manufactured in 2021 [42]. China thus mainly *sold* its vaccines, including to COVAX, nuancing the importance of donations relative to sales in China’s “vaccine diplomacy” [43].

China’s ability to supply vaccines in Africa, where COVAX faltered, appears to have frustrated Western countries, whose major financial contributions and donation pledges to COVAX did not reap similar diplomatic gain. The quest for greater diplomatic influence helps to explain why the EU, in a first instance, donated a substantial share of doses *outside* of the initiative, despite supporting COVAX too. One of the justifications for the EU’s efforts to develop its own “targeted vaccine-sharing” with low-income and neighboring countries was to provide more visibility on the world stage [44]. EU documents state that although to the general COVAX approach means possible soft/regional targeting, "it implies rather limited visibility for member States, Team Europe and the European Union” [45]. The EU subsequently established a system to facilitate dose donations to countries associated with its European Neighborhood Policy, which aims at bringing the EU and its neighbors closer, such as countries from the Eastern partnership region [46]. The effort to persuade donors to channel doses to COVAX instead helps to explain why COVAX appears to have been increasingly willing to accommodate donors’ desire for a degree of earmarking and greater visibility [46].

Despite vaccine diplomacy often being associated with China, we found that virtually every donor country practiced a form of “vaccine diplomacy” in the context of vaccine donations, either through direct bilateral donations, or through COVAX, confirming previous research showing that Western countries, just like China, conceive of global health as a soft power tool. For instance, Gagnon and Labonté [47] concluded that the primary reason the UK integrated health into its foreign policy in the first place was self-interest, including security and economic interests, as well as “a way to enhance UK’s international reputation”. Western countries’ vaccine donations served to brand them as charitable “donors” motivated by global solidarity, distracting from the image civil society groups had painted of them as vaccine hoarders driven by national self-interest [48]. Countries’ frequent violation of COVAX’s dose-sharing principles suggests that they privileged short-term diplomatic recognition over maximizing the impact of their donations. In some senses, what had been a multilateral effort transformed into an opportunity for countries to display global solidarity while allowing them to reap diplomatic and reputational gains.

### Commercial interests

The interplay between national security, diplomatic and commercial interests also compromised the effectiveness of COVAX’s dose-sharing scheme, a dimension often neglected in state-centric analyzes of global health security and global health diplomacy. Although COVAX's dose-sharing principles were addressed to *both* donor countries and vaccine manufacturers, vaccine manufacturers appear to have done little to enable their implementation. They did not donate any vaccine doses to COVAX, committing instead to sell it doses “at cost” through commercial deals. Overall, commercial manufacturers have prioritized the most lucrative markets and, when export restrictions were imposed, domestic markets [48]. This was for instance the case of the Serum Institute of India, which was initially supposed to provide most of COVAX’s supply of AstraZeneca vaccines before an export ban was imposed to prioritize supply to India. In November 2021, Bruce Aylward, senior advisor to the WHO Director General, vocally expressed frustration about the industry’s lack of transparency around delayed deliveries to COVAX: “Show us the queue. Where is the order? And how do you organize your queue? If it is by the order in which they were placed, then we would have been served a long time ago” [44].

We argue that promoting dose-sharing can be seen as a convenient strategy for the pharmaceutical industry to pursue its economic interests. First, instead of prioritizing “at cost” deliveries to COVAX, dose-sharing allows manufacturers to continue deliveries to their most lucrative markets and to maximize profits by selling doses at full price to wealthy countries, who can then share or donate them forward. Second, it enables industry to claim that production has been scaled up sufficiently to meet global demand but must simply be redistributed, deflecting attention from demands for a waiver on intellectual property on vaccines and other Covid-19 diagnostics and therapeutics at the World Trade Organization [49]. Third, it obscures the power the industry holds over the global allocation of vaccines, placing primary responsibility for redistribution (or failure to do so) on wealthy country governments.

When, in May 2021, IFPMA – the global voice or lobby of the pharmaceutical industry – endorsed dose-sharing as essential for vaccine equity, it committed to “immediately work with governments that have significant domestic supplies of Covid-19 vaccine doses to share a meaningful proportion of their doses with low- and lower-middle-income countries in a responsible and timely way through COVAX or other efficient established mechanisms” [50]. Contracts and negotiations over vaccine supply are confidential trade secrets, but media sources suggest that some vaccine companies *impeded* donations through contractual clauses. The New York Times reported in January 2021 that contracts for Covid-19 vaccine purchases prohibited donations [51]. After gaining access to leaked contracts, Vanity Fair reported that the US agreed in contracts with Pfizer, Moderna, AstraZeneca, and Johnson & Johnson (Janssen) that, “the Government may not use, or authorize the use of, any products or materials provided under this Project Agreement, unless such use occurs in the United States” [49]. The report cited government officials acknowledging that manufacturers had refused to negotiate this clause, but were complicit with this practice to secure advantageous conditions for domestic supply [49]. The contracts had to be renegotiated before doses could be sent abroad, due to liability issues: in case of an adverse side effect, who should be responsible? The buyer/donor? The manufacturer? Or the recipient country?

COVAX foresaw this “bottleneck” and offered a no-fault compensation scheme to cover cross-border liability hurdles. This COVAX liability scheme only covers doses directly sent from the manufacturing plant – i.e., that have not been delivered to the purchasing country. This partly explains why several countries that pledged to donate doses through COVAX channeled doses bilaterally instead. Despite COVAX’s offers, it reportedly took many months before manufacturers agreed to terms that would enable vaccine donations; 6 months or longer for separate agreements between the EU and Pfizer, Johnson & Johnson, and Moderna, resulting in agreements only in the third quarter of 2021 [52, 53]. The Norwegian diplomat who led the negotiations with Pfizer stated that “negotiations were more complicated than had been expected” [53].

Meanwhile, Germany wrote to the EU in late October 2021 to express frustration over “ongoing bureaucratic, logistical and legal problems imposed by vaccine makers on EU countries wanting to donate surplus shots”, which impede donations and may lead to wasting of vaccine doses [54]. For example, Moderna only agreed to terms that made it possible for the EU to donate 70 million vaccine doses through COVAX in mid-November 2021 [52]. These contractual impediments and delays raise questions about the degree of industry’s commitment to dose-sharing, and expose public officials’ failure to anticipate the extent and complexity of the legal impediments. The EU correctly claimed in 2020 that its APA with Moderna ensured that “member States can also decide to donate the vaccine to lower and middle-income countries or to re-direct it to other European countries” [55] but this applied only to bilateral donations. To share procured doses with COVAX it had to negotiate complementary agreements with individual manufacturers [53].

At the same time, that donor governments do not publicly challenge these inconsistencies may reflect that they have a self-interest in accommodating industry requirements, whether to ensure priority access and favorable terms for their own citizens, or to reap tax benefits from manufacturers’ profits. BioNTech, for example, is estimated to boost Germany’s Gross Domestic Product (GDP) by 0.5% in 2021 [56], will create jobs, and will generate important tax revenues – €3.1 billion for the first months of 2021 alone [57]. This may partly explain Germany’s solidarity with the pharmaceutical industry in opposing a waiver on their intellectual property rights.

### COVAX and the limits of the ‘super PPP’ model to solve global health crises

COVAX’s leaders and supporters tend to blame “external factors” for the problems the initiative has encountered, while highlighting its pragmatism and flexibility to deliver vaccines despite hurdles [58]. Indeed, key partners did not play by the rules – or to be precise, the voluntary principles they committed to when joining COVAX – thereby undermining the initiative’s work. In addition, some countries (China, India, and Russia) did not contribute to COVAX’s dose-sharing and the US only joined the initiative late, under the Biden administration. However, many of the problems revealed by our analysis are home grown from within COVAX and derive from the initiative’s governance and institutional design, which limits the leverage that COVAX has to shape the behavior of donor states and the pharmaceutical industry.

First, and as we have argued elsewhere, the initiative was set-up as a “super-PPP” that tried to accommodate the divergent interests of its donors, suppliers, and recipients, resulting in an incredibly complex governance structure. This complexity blurs lines of accountability [13]. The boards of the respective participating institutions formally bear the ultimate responsibility for the organizations’ actions, but have no mandate for the overall COVAX initiative and these boards are dominated by donor governments and the pharmaceutical industry [59] – the same partners that undermined the initiative’s procurement efforts by pursuing their own self-interests. Donor governments and the industry have been able to wield power within the initiative to obtain the conditions they want, even when this was at odds with the goal of maximizing vaccine equity. The fact that COVAX has no mechanisms for enforcing its dose-sharing principles shows that the initiative’s institutional design is poorly suited to the challenges it faces. In the absence of other enforcement mechanisms, COVAX’s leaders continue to insist on voluntary principles. A year after publishing its dose-sharing principles, COVAX issued a joint statement with the African Union and the Africa Centre for Disease Control outlining a series of strikingly similar standards for dose donations, again “calling on” donors and manufacturers to “commit to this effort by adhering to the following standards, beginning from 1 January 2022” [60].

COVAX’s structure as a super-PPP has also enabled a critical lack of transparency. The lack of transparency relating to contracts with pharmaceutical companies, vaccine prices, subsidies for Research & Development and scaling up production capacity, etc. have been widely noted, and applies to dose-sharing too [61]. The case of earmarking vaccine donation is a clear example. Despite considerable efforts (see methods), we were unable to establish how the COVAX Facility reached its decision to permit earmarking, directly at odds with its stated principles. There is no mention of the issue of earmarking in the minutes of Gavi’s board meetings, which oversees the COVAX Facility, or in the publicly available minutes of meetings either of the AMC engagement group or of the Shareholders Council. The COVAX Coordination Meeting may have discussed the issue as it is the highest-level coordinating body within the initiative, but summaries of its discussions are not publicly available. Officials from the COVAX Facility did not answer our repeated enquiries about earmarking.

On the one hand, proponents have argued that without COVAX, vaccine inequity would be even worse [62]. On the other hand, it is problematic that its voluntary public-private partnership model appears to allow a culture of deference to the most powerful partners to keep them engaged. Instead of using naming and shaming tactics to deplore broken donation pledges or inadequate deliveries as it might have done, COVAX’s external communication has systematically praised its suppliers and “generous donors” [63], even when their donation practices complicated its equitable allocation proposals. This is doubly ironic since the donors are the same nation states that directly undermined the initiative’s procurement efforts by signing unilateral deals in 2020 (and beyond) that pushed COVAX to the back of the vaccine supply queue, making its goal of delivering two billion vaccines in 2021 unachievable. COVAX has also been mostly silent about the hurdles imposed by manufacturers. Despite the IAVG noting that “not all expected doses from COVAX advanced purchase agreements (APAs) have been honored by vaccine producers according to contractual obligations” [64], to our knowledge the COVAX Facility has not pursued legal action or other sanctions against the producers, in much the same way as COVAX co-lead CEPI did not sanction Moderna’s breach of its equitable access principles [65]. This contrasts with the EU, which took legal action against AstraZeneca after delays in vaccine deliveries [66].

## Conclusion

COVAX’s pivot from global procurement to dose-sharing hub can be seen as a “win-win-win” solution for COVAX itself (which could claim success by having access to more doses), for donor countries (who could rebrand themselves as charitable donors rather than vaccine “hoarders”), and for the pharmaceutical industry (maintaining the status quo on intellectual property rights and protecting commercial interests). Although dose-sharing helped to address COVAX’s supply challenges and increased its deliveries, the impact of the mechanism was undermined by donors’ and manufacturers’ pursuit of national security, diplomatic and commercial interests through vaccine donations, which COVAX largely accommodated. The lack of transparency and accountability mechanisms within COVAX’s overly complex governance structure enabled these practices. Its powerful narrative of success, in turn, jars with its continued struggle to, in its own words, “accelerate” global vaccine equity. A greater willingness to confront and reckon with COVAX’s governance challenges is urgently needed given ongoing efforts to institutionalize and make the initiative permanent within the global health security governance architecture.

## Methods

The analysis in this article draws on ongoing social scientific research into public-private cooperation in pandemic response focusing on global cooperation on Covid-19 vaccine access, including document review, analysis of COVAX’s governance structure and 26 in-depth interviews with individuals involved in COVAX and its lead organizations Gavi, CEPI and the WHO [13]. Here we describe the specific methods we used to assess the implementation of dose-sharing through COVAX.

### Publicly available databases

We base our quantitative analysis of dose-sharing on data extracted on January 3, 2022, from UNICEF’s “Covid-19 vaccine market dashboard,” the most comprehensive, authoritative, and up-to-date database on vaccine deliveries worldwide [21].^2^ To our knowledge, this database is the only one that provides both first-hand information on dose-sharing with COVAX and includes data about bilateral vaccine donations, drawn from media reports. We extracted data on 1) the total number of donated doses delivered to any country, 2) the number of donated doses delivered through COVAX, 3) the number of donated doses delivered bilaterally, and 4) the three main recipient countries for each channel of donation. We compiled detailed data for 40 countries who donated 1 million doses or more, or donated through COVAX, and grouped the 33 countries who donated less than 1 million doses in a category ‘others’. We provide in Additional file 1 the data extracted from the UNICEF Covid-19 vaccine market dashboard, both for transparency purposes and for archival reasons, as the data is constantly updated and erased. We draw from COVAX’s own overview of countries’ pledges to assess countries’ delivery [28].

### COVAX documents, public communication, media coverage and interviews

To gain a qualitative understanding of COVAX's dose-sharing practices and their implementation, we analyzed documentation made publicly available on the websites of COVAX’s co-hosts, Gavi, the Vaccine Alliance (which is responsible for COVAX’s procurement and dose-sharing mechanism) and the WHO.^3^ We reviewed documentation presented at Gavi’s last four board meetings (December 2020–November 2021); meeting summaries of the COVAX AMC Engagement Group and Shareholders Council; and reports of the IAVG, which monitors COVAX's allocation proposals. We also reviewed the 20 press releases regarding dose-sharing that Gavi published between April and November 2021; Gavi’s Twitter feed between April and November 2021, extracting all the tweets about COVAX’s donations; and press reports on dose-sharing/vaccine donation in leading international newspapers and periodicals (for example The New York Times, Politico, The Guardian and Le Monde); reports in scientific journals (e.g. The Lancet); and specialist online reporting sites (e.g. Geneva Health Files, Development Today, Devex).

We were unable to find reliable and precise information about the extent of earmarking of doses shared with COVAX in the public databases mentioned. Despite a dozen emails sent to COVAX and to individuals involved in relevant working groups, we received no answers to our questions about earmarking, or to our requests for interviews to discuss dose-sharing specifically - except from two individuals not directly involved in the COVAX Facility. We also draw on data obtained through a freedom of information request to the Norwegian Agency for Development Cooperation (Norad).

## Supplementary Information

**Additional file 1.** Database. Source: data extracted from the UNICEF Covid-19 vaccine market dashboard on January 3rd, 2022 [21].

## Data Availability

The analysis is based primarily on publicly available data. Interview transcripts are only available to the research team, in compliance with the terms of the research approval and GDPR.

## Abbreviations

ACT-A: Access to Covid-19 Tools Accelerator
AMC: Advanced Market Commitment
APA: Advanced Purchase Agreements
CEPI: Coalition for Epidemic Preparedness Innovation
EU: European Union
GDP: Gross Domestic Product
IAVG: Independent Allocation of Vaccine Group
IFPMA: International Federation of Pharmaceutical Manufacturers & Associations
Norad: Norwegian Agency for Development Cooperation
ODA: Official Development Assistance
PPP: Public-private partnership
UK: United Kingdom
UNICEF: United Nations Children’s Fund
US: United States
WHO: World Health Organization

## Footnotes

1 In relation to COVAX, the terms sharing and donating have been used to describe 1) nonbinding *pledges* to share or donate doses in the future (often without a time line or specified deadline); 2) the *transfer of options* to buy (future) doses (by COVAX’s self-financing member countries to the AMC countries); 3) *accepted doses* by COVAX, i.e. firm commitments or *guarantees to donate* or share forthcoming shipment from a manufacturer; 4) donations of *doses that are actually released to COVAX and delivered* to specific countries, and 5) *facilitated doses,* which are doses purchased by a donor on behalf of COVAX (fully paid for, or whose costs are shared between the donor and COVAX). It should be noted that doses delivered does not equate with the number of individuals vaccinated, since most of the vaccines that are donated require at least two doses to offer significant protection against serious disease and death.

2 We initially extracted the data on November 10, 2021; and updated it after peer review on January 3, 2022 to cover the entire year of 2021. The updated data did not lead to major changes to our results or analysis, but enabled us to nuance some findings.

3 CEPI is also a co-host, but is responsible for vaccine Research & Development, rather than procurement or dose-sharing.
